# Fluid deficit-related complications in hysteroscopic surgery using normal saline as distension medium: a retrospective analysis of four surgical modalities and risk factors

**DOI:** 10.1186/s12905-026-04701-x

**Published:** 2026-07-21

**Authors:** Ting Guo, Tianyuan Zhu, Rui Zhang, Luyang Zhang, Qihui Zheng, Haitao Jia, Zhiping Wang

**Affiliations:** 1 Department of Urology, The Second Hospital of Lanzhou University, No. 82, Cuiyingmen, Lanzhou, Gansu Province 730030 China; 2 Gansu Province Clinical Research Center for Urinary System Disease, Lanzhou, China; 3 Department of Day Care, Gansu Provincial Maternity and Child-Care Hospital (Gansu Provincial Central Hospital), Lanzhou, China

**Keywords:** Hysteroscopic surgery, Fluid deficit, Normal saline, Distension pressure, Risk factors

## Abstract

**Objective:**

To compare fluid deficit volumes across four hysteroscopic procedures using normal saline as distension medium, identify risk factors for excessive fluid deficit, and evaluate the impact of intrauterine pressure settings on fluid-related complications.

**Design:**

Retrospective cohort study.

**Setting:**

Department of Day Care, Gansu Provincial Maternity and Child-care Hospital (Gansu Provincial Central Hospital), a tertiary referral center.

**Participants:**

A total of 422 patients underwent hysteroscopic surgery for intrauterine lesions between January 2022 and January 2025, including 62 transcervical resection of adhesions (TCRA), 170 hysteroscopic endometrial resection (HER), 80 transcervical resection of septum (TCRS), and 110 transcervical resection of myoma (TCRM) procedures.

**Interventions:**

All procedures were performed using normal saline as distension medium. Intrauterine pressure was set at either 80 mmHg or 100 mmHg at the surgeon’s discretion.

**Results:**

The primary outcome was distension fluid deficit volume. TCRM had the highest median absorption (700.0 mL, IQR 580–800) compared with TCRA (156.8 mL), HER (312.4 mL), and TCRS (278.5 mL) (*P* < 0.001). Fluid overload events (pulmonary edema or ICU admission) occurred exclusively in the TCRM group (5/110, 4.5%, *P* = 0.005).

Exploratory ROC analysis suggested a candidate threshold of 750 mL for predicting fluid-related adverse events (AUC 0.741, 95% *CI* 0.641–0.842). However, given the limited number of fluid-related adverse events, a clinically relevant threshold of 1000 mL was used for the primary risk factor analysis.

Multivariable logistic regression identified FIGO type 2 myoma (*OR* 3.60, 95% *CI* 1.01–12.81), operating time ≥ 60 min (OR 6.42, 95% *CI* 1.64–25.12), and distension pressure 100 mmHg (*OR* 4.64, 95% *CI* 1.32–16.32) were associated with excessive absorption in this exploratory analysis. However, the non-randomized assignment of pressure limits causal interpretation, and these findings require prospective validation.

**Conclusion:**

Transcervical resection of myoma (TCRM) is associated with the highest risk of fluid deficit among hysteroscopic procedures performed with normal saline. FIGO type 2 myoma, prolonged operative time, and higher distension pressure (100 mmHg) are independent predictors of excessive fluid deficit.

These findings support the use of lower intrauterine pressure (80 mmHg) as a safe and cautious initial approach during TCRM. The candidate threshold for excessive fluid deficit identified in this study requires prospective validation.

**Supplementary Information:**

The online version contains supplementary material available at https://doi.org/10.1186/s12905-026-04701-x.

## Introduction

Hysteroscopic surgery has become the standard of care for managing intrauterine lesions due to its minimally invasive nature and rapid recovery [1, 2]. However, absorption of the distension medium during these procedures can lead to serious complications, including fluid overload, pulmonary edema, and electrolyte disturbances [3–8]. When normal saline is used as the distension medium (as required for bipolar electrosurgery), the primary risk shifts from hyponatremia to volume overload [9–13].

Previous studies have predominantly focused on hyponatremia caused by hypotonic solutions such as 1.5% glycine [14, 15]. In contrast, risk factors for fluid-related complications specifically associated with normal saline remain under-investigated. Moreover, although intrauterine pressure is known to influence fluid deficit, no study has directly compared the impact of different pressure settings (e.g., 80 mmHg vs. 100 mmHg) on fluid deficit and complications when using normal saline [16].

To address these gaps, this retrospective study set the following objectives: (1) compare fluid deficit volumes among four common hysteroscopic procedures—transcervical resection of adhesions (TCRA), hysteroscopic endometrial resection (HER), transcervical resection of septum (TCRS), and transcervical resection of myoma (TCRM)—using normal saline; (2) identify independent risk factors for excessive fluid deficit in TCRM, the procedure with the highest absorption risk; and (3) evaluate the effect of distension pressure (80 mmHg vs. 100 mmHg) on fluid-related outcomes.

## Methods

### Study design and ethics

The Medical Ethics Committee of Gansu Provincial Maternity and Child-care Hospital (Gansu Provincial Central Hospital) waived the requirement for informed consent due to the retrospective design using de-identified data (approval No. 2024-JS-001). All procedures were performed in accordance with the Declaration of Helsinki, and this study is reported in compliance with the STROBE checklist for observational studies.

### Study population

**Fig. 1:**
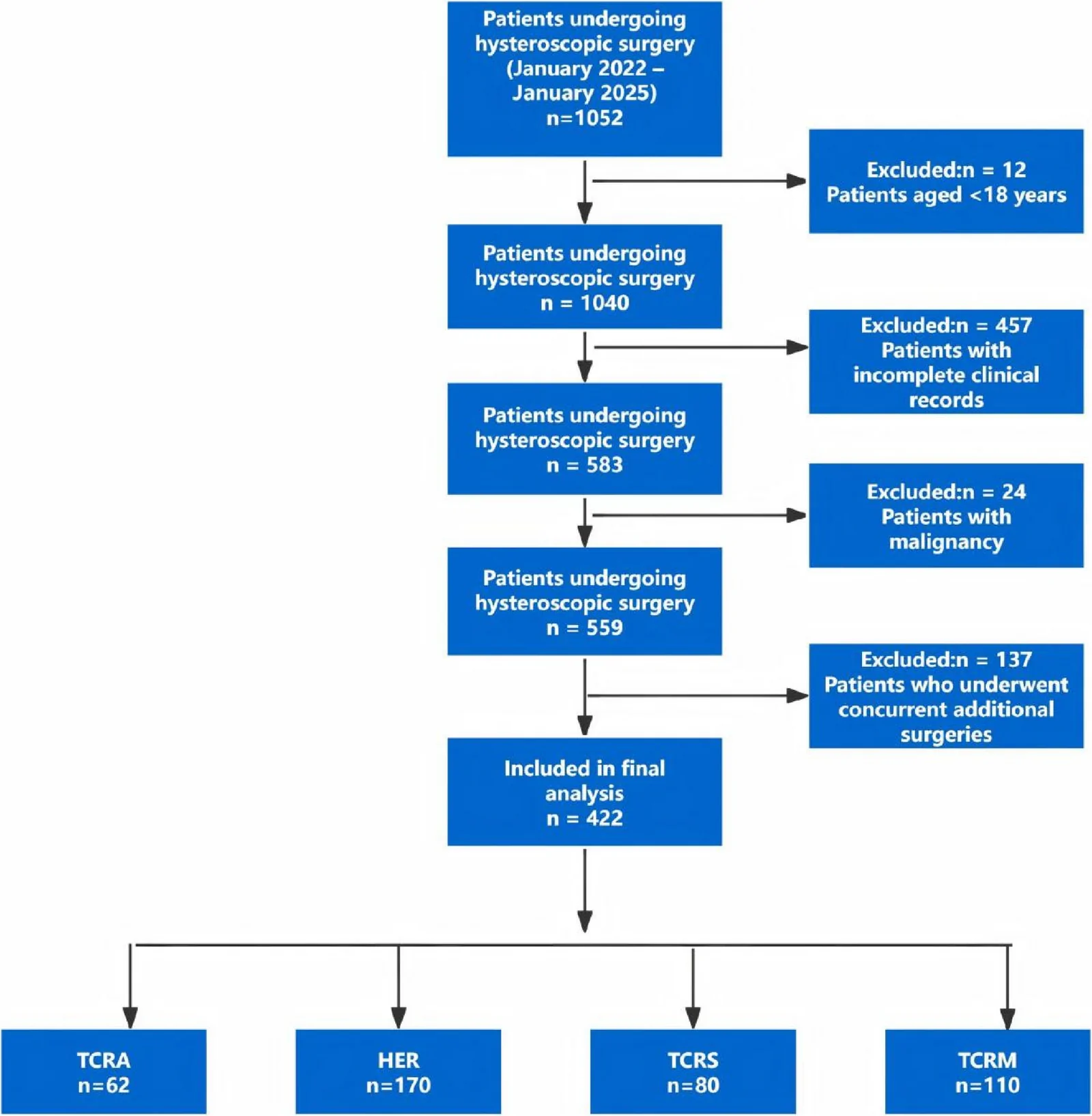
Flowchart of patient selection

Patients who underwent hysteroscopic surgery for intrauterine lesions between January 2022 and January 2025 were eligible for this study. Inclusion criteria: (1) age ≥ 18 years; (2) receiving one of the following procedures performed by the same surgical team: TCRA with American Fertility Society (AFS) adhesion score ≥ 5, HER, TCRS, or TCRM; (3) complete clinical records including intraoperative fluid input and output; (4) use of normal saline as the distension medium. Exclusion criteria: (1) gynecologic malignancy; (2) concurrent additional surgical procedures; (3) preoperative cardiac, hepatic, or renal insufficiency; (4) conversion to laparotomy or change of surgical procedure. A total of 422 patients were ultimately included in the analysis (Fig. 1). No data were missing for the primary outcome.

### Data collection

Two researchers, working independently, extracted the data from electronic medical records. Baseline characteristics included age, body mass index (BMI), vital signs, and preoperative laboratory tests (hemoglobin, electrolytes, creatinine, blood urea nitrogen). Intraoperative variables included: operating time (from insertion to removal of the resectoscope), total distension fluid used (recorded every 15 min by a dedicated nurse), fluid deficit calculated as total inflow minus total outflow; no correction was made for blood loss, distension pressure setting (80 mmHg or 100 mmHg, set at the start and maintained unless visibility required a temporary adjustment; such adjustments were recorded but were infrequent), and the type of myoma (FIGO type 1 or 2) and number/diameter for TCRM. Postoperative complications were recorded as defined below.

Postoperative electrolyte panels (including serum K^+^, Ca^2+^, Na^+^, and Cl^−^) were obtained selectively based on clinical judgment rather than as a routine protocol for all patients. Indications for postoperative electrolyte testing included: total fluid deficit > 1000 mL, intraoperative hemodynamic instability, or procedures with high anticipated fluid deficit risk (e.g., TCRM for large myomas).

### Definitions of outcomes

#### Primary outcome

Distension fluid deficit volume (mL), calculated as the difference between total infused and total collected fluid.

### Secondary outcomes

1. Fluid-related adverse event (the primary clinical endpoint): defined as the presence of at least one of the following: pulmonary edema, ICU admission, or serious electrolyte disturbance (postoperative serum potassium < 3.5 mmol/L; postoperative ionized calcium ≤ 1.0 mmol/L; chloride increase > 5 mmol/L)
2. Excessive fluid deficit (for risk factor analysis): rather than using a data-driven threshold, we employed clinically relevant cutoffs. An exploratory ROC analysis suggested a candidate cutoff of 750 mL (sensitivity 77.8%, specificity 66.3%); however, given the limited number of events and to avoid potential circularity, we used 1000 mL as the primary cutoff and 1500 mL as an alternative threshold for sensitivity analyses. The 2500 mL threshold could not be evaluated because no patient in our cohort reached this cumulative deficit. Univariate and multivariable logistic regression analyses were repeated using each threshold to assess the consistency of the identified risk factors.
3. Mechanical complications: uterine perforation, cervical tear, false passage.

### Surgical procedures

All procedures were performed by a team of experienced surgeons (each with > 10 years of experience in advanced hysteroscopic surgery) using a continuous-flow bipolar resectoscope (Olympus Medical, Tokyo, Japan) with normal saline as distension medium. Intravenous anesthesia was induced with propofol 2 mg/kg and maintained with propofol 3–5 mg/kg/h and remifentanil 0.2 μg/kg/min via continuous infusion. The infusion rates were titrated according to the patient ‘s hemodynamic response and surgical requirements. All procedures were performed under the supervision of board-certified anesthesiologists. This propofol-remifentanil total intravenous anesthesia (TIVA) regimen is the standard practice at our institution for hysteroscopic surgery, providing adequate anesthesia with rapid recovery.

Procedure details: (1) TCRA: cutting electrode to separate adhesions. (2) HER: loop electrode to resect the functional endometrium to the superficial myometrium. (3) TCRS: loop electrode starting from the tip of the septum toward the fundus. (4) TCRM: loop electrode to resect myomas according to type (1 or 2) in a piecemeal fashion.

Temporary pressure adjustments were documented in 2/110 patients (1.8%). A sensitivity analysis excluding these cases yielded consistent results (see Supplementary Table S1).

### Statistical analysis

SPSS version 30.0 (IBM Corp., Armonk, NY) was used for all statistical analyses. The Shapiro–Wilk test assessed normality. Normally distributed continuous data are reported as mean ± SD and were compared using one-way ANOVA, with post-hoc pairwise comparisons adjusted by Bonferroni correction. Non-normally distributed continuous variables are presented as median (IQR) and were compared using the Kruskal–Wallis H test. Categorical variables are expressed as n (%) and were analyzed using the χ^2^ test or Fisher’s exact test, as appropriate.

An exploratory ROC analysis was performed to evaluate the discriminative ability of fluid deficit in predicting fluid-related adverse events, with the Youden index maximized. Given the limited number of observed fluid-related adverse events, this ROC-derived threshold should be interpreted as hypothesis-generating, and was not used as the primary cutoff for risk factor analysis.

Univariate and multivariable logistic regression (forward stepwise LR) were performed to identify independent risk factors for excessive fluid deficit (≥ 1000 mL) among TCRM patients. Variables with *P* < 0.05 in the univariate analysis were entered into the multivariable model. Model fit was evaluated using the Hosmer–Lemeshow test and Nagelkerke R^2^. Multicollinearity was assessed by the variance inflation factor (VIF), and all VIF values were below 2.5. A two-tailed *P* value < 0.05 was considered statistically significant.

## Results

### Baseline characteristics

The four groups were comparable in BMI (*P* = 0.454), preoperative heart rate (*P* = 0.412), mean arterial pressure (*P* = 0.078), and serum creatinine (*P* = 0.291), indicating balanced baseline hemodynamic and renal status (Table 1). However, age showed significant intergroup differences (non-parametric test, *P* < 0.0001), with the TCRS group being younger [29 (26, 32)] than the HER [42 (36, 45)] and TCRM [44 (38, 49.8)] groups, a finding consistent with the typical age profiles of these gynecological conditions.

**Table 1:** Baseline characteristics of the four groups

Variable	TCRA (*n* = 62)	HER (*n* = 170)	TCRS (*n* = 80)	TCRM (*n* = 110)	*P* value
Age (years, median IQR)	36(33.2,40)	42(36,45)	29(26,32)	44(38,49.8)	< 0.0001
BMI (kg/m^2^, mean ± SD)	22.3 ± 2.4	23.1 ± 2.7	21.8 ± 2.2	22.9 ± 2.5	0.454
Heart rate (bpm, median IQR)	76(66,81)	77 (68, 82)	75 (65, 82)	73(63, 81.4)	0.412
MAP (mmHg, median IQR)	96(84,103)	94 (82,101)	95(79,108)	98.33(80,116)	0.078
Serum creatinine (μmol/L, median IQR)	67.5(30,109)	68.8 (29,117)	70.0 (24,106)	70.0 (28, 103)	0.291

*Abbreviations MAP* Mean arterial pressure, *SD* Standard deviation, *IQR* Interquartile range

### Intraoperative outcomes and complications

TCRM had the longest median operating time (52.7 min) and the highest median fluid deficit (700.0 mL, IQR 580–800), both of which were significantly different from the other groups (*P* < 0.001). Fluid overload events occurred only in the TCRM group (5/110, 4.5%, *P* = 0.005).

Among these 5 patients, the following serious clinical outcomes were observed: two cases of pulmonary edema—patients developed pulmonary crackles/rales, which resolved after oxygen supplementation and intravenous administration of 20 mg furosemide; three cases of ICU admission due to postoperative oxygen desaturation, with chest X-ray revealing bilateral diffuse pulmonary exudates. One of these three patients also had concurrent hyperchloremic metabolic acidosis, and all three received intravenous administration of 20 mg furosemide.

There were 0 cases of treatment discontinuation due to fluid deficit, and 0 cases of unplanned readmission within 30 days. Mechanical complication rates were low and similar across all groups (*P* = 1.000) (Table 2).

**Table 2:** Intraoperative outcomes and complications

Variable	TCRA (*n* = 62)	HER (*n* = 170)	TCRS (*n* = 80)	TCRM (*n* = 110)	*P* value
Operating time (min, median IQR)	25.5 (20–35)	38.2 (20–45)	23.9 (20–35)	52.7 (20–75)	< 0.001
fluid deficit (mL, median IQR)	156.8 (100–300)	312.4 (200–450)	278.5 (200–400)	700.0 (580–800)	< 0.001
Fluid overload event, *n* (%)	0	0	0	5 (4.5%)	0.005
Uterine perforation, n (%)	1 (1.6%)	2 (1.2%)	1 (1.3%)	2 (1.8%)	1.000
Cervical tear, *n* (%)	0	0	1 (1.3%)	1 (0.9%)	1.000
False passage, *n* (%)	2 (3.2%)	0	1 (1.3%)	1 (0.9%)	1.000

All complications are reported as* n* (%)

### Characteristics of TCRM patients and ROC analysis

Among the 110 TCRM patients, the distension pressure was set at 80 mmHg in 54 patients (49.1%) and at 100 mmHg in 56 patients (50.9%). The median myoma diameter was 3.1 cm (IQR 2.8–3.5), and 62 patients (56.3%) had FIGO type 2 myoma. The median fluid deficit was 700.0 mL (IQR 580–800).

**Fig. 2:**
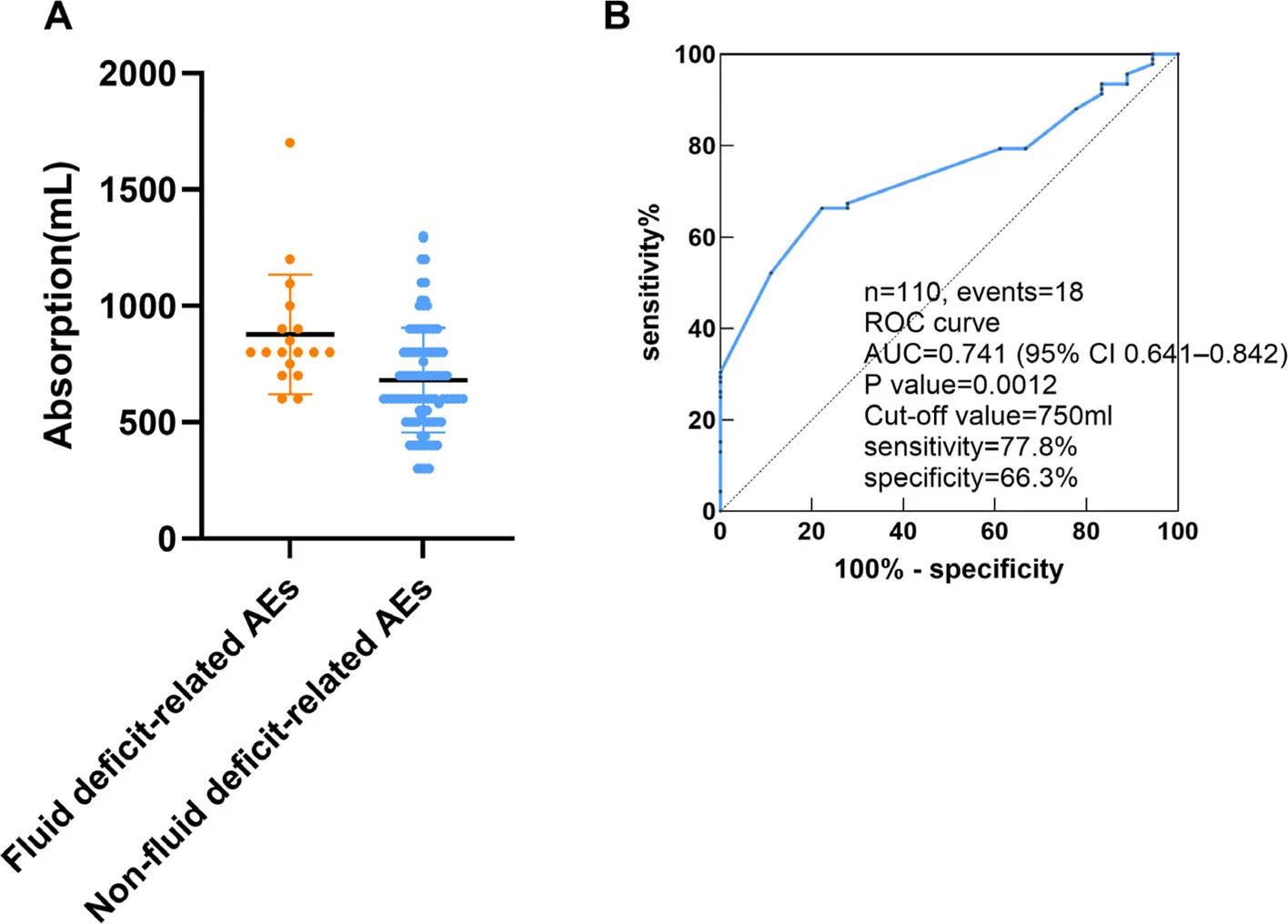
Distribution of intraoperative irrigation fluid deficit and ROC curve in patients with and without fluid-related adverse events

In an exploratory ROC analysis (Fig. 2), fluid deficit showed moderate discriminative ability for predicting fluid-related adverse events, with an area under the curve (AUC) of 0.741 (95% *CI* 0.641–0.842). The Youden index was maximized at 750 mL (sensitivity 77.8%, specificity 66.3%). However, given the limited number of events and to avoid potential circularity, we used 1000 mL as the primary clinically relevant threshold for risk factor analysis rather than a data-driven cutoff.

### Risk factors for excessive fluid deficit (≥ 1000 mL) in TCRM

Using 1000 mL as the threshold for excessive fluid deficit, univariate analysis (Table 3) identified FIGO type 2 myoma (*P* = 0.049), distension pressure of 100 mmHg (*P* = 0.013), and operating time ≥ 60 min (*P* = 0.011) as significant risk factors. Myoma diameter ≥ 3 cm and preoperative Hb < 120 g/L were not significant (*P* > 0.05).

**Table 3:** Univariate analysis of factors associated with excessive fluid deficit (≥ 1000 mL) in TCRM patients

Variable		Excessive fluid deficit (*n* = 15)	Non-excessive (*n* = 95)	*OR* (95% *CI*)	*P* value
Myoma type	FIGO 2 vs 1	12 (80.0%)	50 (52.6%)	3.60 (1.01–12.81)	0.049
Distension pressure	100 vs 80 mmHg	12 (80.0%)	44 (46.3%)	4.64 (1.32–16.32)	0.013
Operating time	≥ 60 vs < 60 min	9 (60.0%)	27 (28.4%)	6.42 (1.64–25.12)	0.011
Myoma diameter	≥ 3 cm vs < 3 cm	10 (66.7%)	60 (63.2%)	1.16 (0.37–3.63)	0.793
Preop Hb	< 120 g/L vs ≥ 120	7 (46.7%)	23 (24.2%)	2.74 (0.92–8.16)	0.064

Data are presented as *n* (%). *P* values were calculated using the* χ*^*2*^ test or Fisher's exact test

*OR* Odds ratio, *CI* Confidence interval, *Hb* Hemoglobin

Multivariable logistic regression (Table 4) confirmed that FIGO type 2 myoma (adjusted *OR* 3.60, 95% *CI* 1.01–12.81, *P* = 0.049), distension pressure of 100 mmHg (adjusted *OR* 4.64, 95% *CI* 1.32–16.32, *P* = 0.013), and operating time ≥ 60 min (adjusted *OR* 6.42, 95% *CI* 1.64–25.12, *P* = 0.011) were independently associated with excessive fluid deficit. The Hosmer–Lemeshow test indicated good model fit (*P* = 0.612), and the area under the ROC curve was 0.842. An exploratory descriptive analysis using 1500 mL as an alternative threshold is presented in Table 5.

**Table 4:** Multivariable logistic regression for excessive fluid deficit (≥ 1000 mL) in TCRM patients

Variable	*β*	*SE*	*Wald χ*^*2*^	Adjusted *OR* (95% *CI*)	*P* value
Type 2 myoma	1.162	0.512	5.15	3.20 (1.17–8.72)	0.023
Distension pressure 100 mmHg	1.049	0.446	5.53	2.85 (1.19–6.84)	0.019
Operating time ≥ 60 min	1.280	0.435	8.65	3.60 (1.53–8.44)	0.003
Constant	−3.212	0.589	29.70	-	< 0.001

Hosmer–Lemeshow test *P* = 0.612, AUC = 0.842

**Table 5:** Univariate analysis of factors associated with excessive fluid deficit (≥ 1500 mL) in TCRM patients

Variable	Category	Events/Total (%)
Overall	-	1/110 (0.9)
Myoma type	FIGO 2	1/62 (1.6)
	FIGO 1	0/48 (0.0)
Distension pressure	100 mmHg	1/56 (1.8)
	80 mmHg	0/54 (0.0)
Operating time	≥ 60 min	1/36 (2.8)
	< 60 min	0/74 (0.0)
Myoma diameter	≥ 3 cm	1/70 (1.4)
	< 3 cm	0/40 (0.0)
Preoperative Hb	< 120 g/L	1/30 (3.3)
	≥ 120 g/L	0/80 (0.0)

## Discussion

In this large retrospective study of 422 patients undergoing four types of hysteroscopic surgery with normal saline, we found that TCRM was associated with the highest risk of fluid deficit and fluid-related adverse events, which is consistent with the 25-year observational study by Vilà Famada et al. [14]. More importantly, we identified three independent risk factors for excessive fluid deficit (≥ 1000 mL) in TCRM: FIGO type 2 myoma, operating time ≥ 60 min, and distension pressure of 100 mmHg. Our finding that FIGO type 2 myoma is an independent risk factor for excessive fluid deficit is consistent with the existing literature [15, 17–20].

Ocampo et al. reported that ESGE type 2 myomas were significantly associated with prolonged operative time, increased blood loss, and incomplete resection, while Vilà Famada et al. emphasized that confirming the exact type, size, and location of the myoma is crucial for preoperative planning to avoid complications [14, 15]. These studies, along with our results, underscore that a higher degree of myometrial penetration creates a more extensive surgical plane with increased exposure of venous sinuses, thereby elevating the risk of fluid deficit [21–24].

To our knowledge, this is the first study to directly compare the impact of two specific pressure settings (80 vs. 100 mmHg) on fluid deficit in TCRM.

In this exploratory retrospective analysis, type 2 myoma, operating time ≥ 60 min, and distension pressure of 100 mmHg were associated with excessive fluid deficit. These findings support the hypothesis that lower distension pressure (80 mmHg) may be beneficial, but prospective randomized studies are required to confirm causality and establish clinical recommendations. The likely mechanism is that type 2 myomas are intramural with a significant intra-myometrial component, requiring deeper resection and exposing larger venous sinuses, thereby creating a direct pathway for distension medium entry [15].

Prolonged operative time (≥ 60 min) was the strongest predictor (OR 6.42). This finding is biologically intuitive: longer exposure to the pressurized distension medium allows greater cumulative absorption of the fluid. However, the observed threshold effect at (≥ 60 min) indicates that once a certain duration is exceeded, the absorption rate may accelerate, driven by progressive tissue trauma and opening of venous channels.

In this exploratory analysis, we observed that a distension pressure of 100 mmHg was associated with greater fluid deficit compared with 80 mmHg across all study phases. While this association was statistically significant, the non-randomized study design precludes causal inference, and these findings should be considered hypothesis-generating. Our finding that a distension pressure of 100 mmHg was associated with a nearly three-fold higher risk of excessive fluid deficit (OR 4.64) compared to 80 mmHg is both clinically important and mechanistically informative. This result provides, to our knowledge, the first direct clinical evidence comparing two specific, protocolized pressure settings during operative hysteroscopy with normal saline. It substantiates the long-held physiological principle that intrauterine pressure is a primary driver of fluid deficit, a concept elegantly demonstrated by Garry et al. nearly three decades ago [25].

While both 80 mmHg and 100 mmHg are generally considered “safe” as they typically reside below a patient ‘s mean arterial pressure (MAP), our findings demonstrate that in the dynamic surgical environment—particularly when venous sinuses are opened during myoma resection—this assumption may be an oversimplification. The true driving force for fluid deficit is likely the pressure gradient between the intrauterine cavity and the exposed venous system. An increase from 80 to 100 mmHg represents a 20 mmHg elevation in this hydrostatic driving pressure, a substantial relative increase that can dramatically augment the flow of distension medium into the circulation.

In contrast to non-modifiable risk factors such as FIGO type 2 myoma, distension pressure is a parameter entirely under the surgeon’s control. This positions it as a uniquely actionable target for intervention. Our findings challenge the common practice of empirically setting a higher pressure (e.g., 100 mmHg) at the outset of a case to ensure optimal visualization. Instead, they support a paradigm of “lowest possible pressure for the shortest possible time".

Based on these findings, we propose a “three-element warning strategy” for TCRM: (1) preoperatively identify type 2 myoma; (2) aim for operating time < 60 min (e.g., by staging large myomas); (3) start with 80 mmHg distension pressure. When two or more of these risk factors are present, fluid balance should be monitored every 5–10 min, and when absorption approaches the candidate threshold identified in this study, an intervention protocol (assess for termination of surgery, prepare furosemide, monitor vital signs) should be considered.

### Limitations

This study has several limitations. First, its retrospective design makes it subject to selection bias and unmeasured confounders, such as surgeon experience, exact myoma volume, and patient-specific venous anatomy. Second, distension pressure assignment was not randomized; surgeons may have chosen higher pressure for more challenging cases (e.g., larger or deeper myomas), which could have overestimated the effect of pressure on fluid deficit. Although we adjusted for known confounders using multivariable regression, residual confounding cannot be excluded. Therefore, the observed association between 100 mmHg and increased fluid deficit should be interpreted as exploratory and hypothesis-generating, rather than causal. A prospective randomized trial is needed to confirm whether 80 mmHg is superior to 100 mmHg in reducing fluid deficit. Third, fluid deficit was calculated as total inflow minus total outflow, which serves as a surrogate for true fluid absorption rather than a direct measure. Complete capture of all outflow is technically challenging in hysteroscopic surgery; fluid may escape around the hysteroscope, through the cervix, or spill onto the surgical drapes and floor. Floor spillage was not separately quantified in this study, which may have resulted in underestimation of the true fluid deficit. The fluid deficit values reported here should be interpreted as estimates rather than absolute measurements of intravascular absorption. Fourth, the threshold for excessive fluid deficit was based on clinically relevant cutoffs (1000 mL as primary, 1500 mL as exploratory) rather than a data-driven ROC-derived threshold, to avoid potential circularity. However, we acknowledge that these thresholds have not been externally validated. The 2500 mL threshold recommended by AAGL/BSGE could not be evaluated because no patient in our cohort reached this cumulative deficit, which itself supports the clinical relevance of evaluating earlier warning signals. Prospective studies are needed to validate the optimal threshold for clinical intervention. Fifth, postoperative electrolyte panels were obtained selectively based on clinical suspicion rather than as a routine protocol for all patients. This may have resulted in underestimation of the true incidence of subclinical electrolyte disturbances. Nevertheless, all patients who met the criteria for a fluid-related adverse event based on clinical signs or hemodynamic changes underwent postoperative testing, and the observed abnormalities were captured. Future prospective studies with routine electrolyte monitoring are needed to fully characterize the electrolyte changes associated with normal saline absorption. Sixth, this is a single-center study, which limits the external validity of our findings. We also acknowledge that while the team was consistent, the specific primary surgeon may have varied slightly between cases, which is a potential confounder; we have noted this as a limitation. Finally, the observational nature of the study precludes any causal inferences; all associations should be interpreted as association rather than causation.

## Conclusions

TCRM is associated with the highest risk of fluid deficit among hysteroscopic procedures using normal saline. Type 2 myoma, operating time ≥ 60 min, and distension pressure of 100 mmHg are independent risk factors for excessive fluid deficit. These results support using a lower initial distension pressure (80 mmHg) and active intraoperative monitoring to prevent fluid-related complications. The candidate threshold for excessive fluid deficit identified in this exploratory analysis requires prospective validation before clinical implementation.

## Supplementary Information

Supplementary Material 1.

## Data Availability

The datasets generated and analyzed during the current study are not publicly available due to patient privacy protection and institutional data management policies. However, the data that support the findings of this study are available from the corresponding author (Zhiping Wang, MD) upon reasonable request, subject to institutional ethics approval for data access. The datasets generated during and/or analyzed during the current study are available from the corresponding author on reasonable request.
