# Supplementary material for: Fluid deficit-related complications in hysteroscopic surgery using normal saline as distension medium: a retrospective analysis of four surgical modalities and risk factors
Source: BMC Womens Health. 2026 Jul 21;26:530. doi: 10.1186/s12905-026-04701-x (PMC13673915; doi:10.1186/s12905-026-04701-x)
Supplement: Supplementary file 1 [file 12905_2026_4701_MOESM1_ESM.docx]

Supplementary Table S1. sensitivity analysis for temporary pressure adjustments

| Analysis | 80 mmHg group (mL/min) | 100 mmHg group (mL/min) | *P* value |
| --- | --- | --- | --- |
| Primary analysis (all patients, n=110) | 600.0 (500–700) | 755.0 (600–925) | <0.001 |
| Sensitivity analysis (excluding temporary adjustments, n=110-2) | 600.0 (500–700) | 780.0 (600–975) | 1.000 |
